# Identification of reversible and druggable pathways to improve beta-cell function and survival in Type 2 diabetes

**DOI:** 10.1080/19382014.2023.2165368

**Published:** 2023-01-29

**Authors:** Smithamol Sithara, Tamsyn Crowley, Ken Walder, Kathryn Aston-Mourney

**Affiliations:** a School of Medicine, IMPACT, Institute for Innovation in Physical and Mental Health and Clinical Translation, Deakin University, Geelong, Australia; b School of Medicine, Bioinformatics Core Research Facility, Deakin University, Geelong, Australia

**Keywords:** Transcriptomics, gene expression, beta-cell, Type 2 diabetes, pharmacology, gene set enrichment analysis

## Abstract

Targeting β-cell failure could prevent, delay or even partially reverse Type 2 diabetes. However, development of such drugs is limited as the molecular pathogenesis is complex and incompletely understood. Further, while β-cell failure can be modeled experimentally, only some of the molecular changes will be pathogenic. Therefore, we used a novel approach to identify molecular pathways that are not only changed in a diabetes-like state but also are reversible and can be targeted by drugs. INS1E cells were cultured in high glucose (HG, 20 mM) for 72 h or HG for an initial 24 h followed by drug addition (exendin-4, metformin and sodium salicylate) for the remaining 48 h. RNAseq (Illumina TruSeq), gene set enrichment analysis (GSEA) and pathway analysis (using Broad Institute, Reactome, KEGG and Biocarta platforms) were used to identify changes in molecular pathways. HG decreased function and increased apoptosis in INS1E cells with drugs partially reversing these effects. HG resulted in upregulation of 109 pathways while drug treatment downregulated 44 pathways with 21 pathways in common. Interestingly, while hyperglycemia extensively upregulated metabolic pathways, they were not altered with drug treatment, rather pathways involved in the cell cycle featured more heavily. GSEA for hyperglycemia identified many known pathways validating the applicability of our cell model to human disease. However, only a fraction of these pathways were downregulated with drug treatment, highlighting the importance of considering druggable pathways. Overall, this provides a powerful approach and resource for identifying appropriate targets for the development of β-cell drugs.

## GRAPHICAL ABSTRACT

**Figure:**
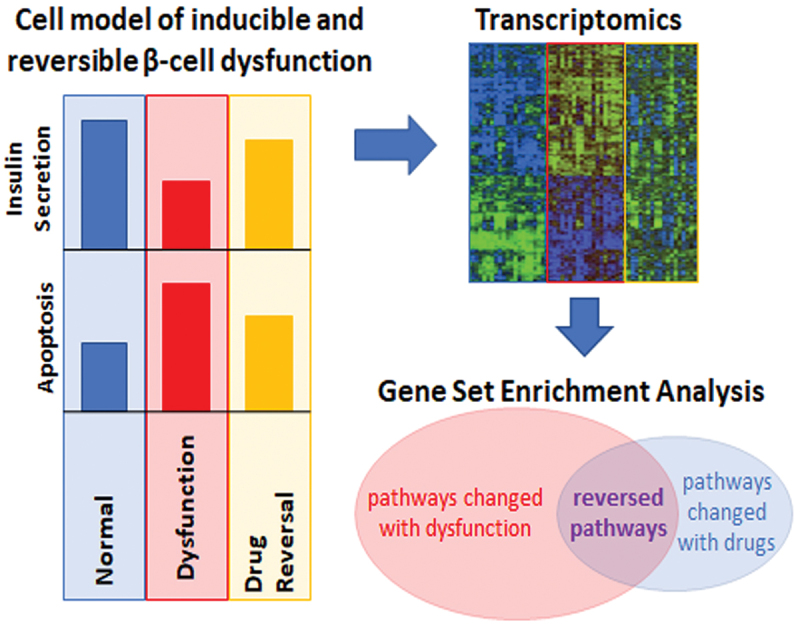

## Introduction

β-Cell dysfunction, a combination of reduced β-cell secretory function and reduced β-cell mass, is an absolute requirement for the development of Type 2 diabetes (T2D).^1^ This dysfunction is progressive, occurring for several years prior to disease diagnosis and throughout the disease. At the time of diagnosis, patients have usually already lost over 50% of their β-cell function and this continues to decline.^1–4^ Along with this decline in function, there is also a continuous decline in β cell mass, mainly due to an increase in apoptosis.^4,5^ Clinically, this manifests as a requirement for additional therapies to maintain adequate glycemic control.^6^ Thus, the most efficient way to treat T2D would be to prevent or delay the onset of the disease by halting or slowing the progression of β-cell failure. Further, as β-cell dysfunction in T2D can be viewed as reversible, at least in the early years of the disease (<10 y),^7^ pharmacological interventions aimed at protecting the β-cells can potentially be used to restore, or at least prevent further loss of, β-cell function in subjects with diagnosed T2D which would result in better patient outcomes.

The increasing prevalence of T2D has led to different approaches in the discovery of new therapeutic targets for the treatment of hyperglycemia. Currently, there are several different drugs available; however, achieving and maintaining long-term glycemic control are still challenging with only 50% of the patients able to meet target glycemic levels.^8,9^ This could be due to the inability of these medications to target the root causes of the disease, especially the progressive decline in β-cell mass and function.^8,10,11^ In fact, no pharmacological agent has yet been shown to be capable of changing the natural course of β-cell dysfunction.^10,12^

Stressors on the β-cell thought to contribute to decreased β-cell function and mass include inflammatory stress, endoplasmic reticulum (ER) stress, metabolic/oxidative stress, amyloid stress and stressed islet integrity.^13^ What is clear is that β-cell failure is not a simple process but involves many different stressors, and despite much research it is not clear which pathway is disrupted first.^13^ However, even though several pathways are involved it has been suggested that given the likely overlap, crosstalk and convergence between pathways, targeting a single molecule could still affect multiple stressor pathways and have beneficial effects on β-cells.^13^ What is common for all of these pathways however is the upstream stressors of increased metabolic load and insulin resistance (demanding increased insulin release)^13^ each of which can be modeled experimentally by inducing chronic hyperglycemic stress. In fact, it has been suggested that initiating therapies aimed against β-cell glucose toxicity in the early stages of the disease will be of the utmost importance in treating and preventing T2D.^14^ It has also been suggested that the appropriate therapy needs to be selected based on the molecular mechanisms of glucose toxicity.^15^ However, given that the full molecular mechanisms of glucose toxicity are complicated and yet to be elucidated, this is very difficult. In addition, while hyperglycemia is bound to result in extensive molecular changes within the β-cell, only some of these changes will be involved in the pathology of β-cell failure. Therefore, identifying which changes are important to target therapeutically remains a challenge.

In complex disease like T2D, there are many genes involved which affect biological function in groups rather than alone. Therefore it may be more effective to understand the signaling pathways involved in the pathological mechanisms rather than individual genes. Other studies have used pathway-based genetic approaches to obtain interesting and relevant pathway outcomes linked to complex diseases such polycystic ovary syndrome, bipolar disorder, coronary artery disease, Crohn’s disease, hypertension, rheumatoid arthritis, Type 1 diabetes, and T2D illustrating the potential of a pathway-based approach in characterizing and identifying novel pathways associated with complex disease.^16–22^

Previous studies have identified pathways associated with T2D by comparing nondiabetic and diabetic subjects.^23–26^ Finding out what pathways are involved in a disease and identifying which of these pathways are affected in each patient may provide more understanding of T2D and could lead to new strategies for diagnosing, treating and preventing disease. However, whether these pathways are altered as a result of the disease or are involved in the pathogenesis of the disease can be difficult to determine. Further, whether these can be effectively targeted pharmacologically, and result in an improvement in the disease state is uncertain. Hence, in this study, we have taken a new approach to this type of analysis. Here we looked at the pathways that are not only changed in a diabetes-like state but are also reversible and can be targeted by drugs. To our knowledge, this is the first study of this kind, which looks at both induction and reversal of dysfunction. This study therefore provides a novel insight into the transcriptional changes accompanying dysfunction of β-cells as well as the successful reversal of dysfunction.

We propose that by using a cocktail of drugs that can successfully treat β-cells undergoing hyperglycemic stress, we can not only prolife the global transcriptomic changes that occur under hyperglycemic conditions but also identify those changes that are reversible pharmacologically. By identifying the pathways that reverse with successful treatment of β-cells, we suggest that this can contribute to target drug design efforts to overcome the current difficulty in identifying drugs that have an overall beneficial effect to protect β-cells in T2D.

## Materials and methods

### Cell treatment

INS 1E cells were treated with Roswell Park Memorial Institute 1640 medium (Gibco Thermo Fisher) supplemented with 10% heat-inactivated fetal bovine serum (Gibco Thermo Fisher), 1% sodium pyruvate, 0.05 mM 2-mercaptoethanol, 1% HEPES and 1% penicillin and streptomycin containing either standard 11.1 mM glucose (as a normal glucose (NG) treatment) or 20 mM glucose (as a high glucose (HG) treatment). It should be noted that a glucose concentration of 11.1 mM is considered normal in culture of β-cells and islets.

For reversal of the HG effects, a cocktail of agents (50 nM Exendin-4, 250 μM metformin and 250 μM sodium salicylate) was added in the final 48 h of the 72-h treatment.

### Cell function

INS-1E cells were first reset to their basal state by incubation for 30 min with 2 mM glucose Krebs–Ringer bicarbonate HEPES (KRBH) buffer. The buffer was then changed to KRBH containing either 2 mM glucose (basal) or 20 mM glucose (stimulated) for 45 min and the supernatant collected for insulin measurement using the Mouse Insulin Ultra-Sensitive ELISA kit (Alpco Diagnostics).^27^ Insulin measurements were normalized to cell number, determined by the CyQUANT Assay (Molecular Probes) as per the manufacturer’s directions.

### Apoptosis

Caspase-3/7 activity was measured using the Caspase-glo 3/7 assay kit (Promega Biosciences) as per manufacturer’s instructions.

### Statistical analysis

Twenty  independent experiments, each with triplicate repeats, was performed. Insulin secretion and apoptosis data were normally distributed and were compared by two-way analysis of variance using least significant difference post hoc analysis with *p* < .05 considered significant.

### RNA extraction, quantification and quality analysis

Treated INS1E cells (*n* = 20) were collected in TRIzol reagent (Invitrogen), RNA extracted by chloroform/ethanol extraction followed by purification using RNeasy Mini columns (Qiagen). The quality and quantity of the RNA were determined using the Agilent Bioanalyzer (Agilent Technologies) and RNA 600 Nano Assay kit (Agilent).^28^ Samples with an RNA Integrity Number (RIN) value ≥7 were deemed sufficient quality for RNAseq (all samples in this study had RIN >9).

### RNAseq

Samples were prepared using the Illumina TruSeq RNA Sample Preparation Kit v2 (Illumina) using TruSeq RNA Sample Preparation v2 LS protocol and 15 indexed adapters as per the manufacturer’s instructions. Verification of the size of PCR enriched fragments was performed using Agilent 2100 Bioanalyzer (Agilent Technologies) and a DNA 1000 chip. To quantify the prepared libraries, qPCR was used according to the Illumina qPCR Quantification Protocol Guide. Indexed DNA samples were normalized to 10 nM and the DNA templates were pooled together for cluster generation in four batches of 15 samples. Next-generation sequencing was then performed using lllumina’s HiSeq 2500 (San Diego).

### RNAseq data analysis

RNAseq data analysis was performed using Illumina BaseSpace cloud computing environment (https://basespace.illumina.com). Quality control of the raw sequence data was performed using the BaseSpace app FastQC Version 1.0.0.^29^ One sample was not of sufficient quality and was excluded from further analysis, while all other samples exceeded the minimum quality value and were well above Q30 (highest quality range). The sequences were trimmed to remove adapter sequences using FASTQ Toolkit Version: 2.0.0 by BaseSpace Labs^30^ and then aligned against the reference genome (Rattus norvegicus UCSC rn5 (RefSeq gene annotations)) using TopHat Alignment app by Illumina.^31^ Quantification or Fragments Per Kilobase of transcript (FPKM) estimation of the reference genes and transcripts were performed using Cufflinks 2.^32^ The data discussed in this publication have been deposited in NCBI’s Gene Expression Omnibus^33^ and are accessible through GEO Series accession number GSE218540 (https://www.ncbi.nlm.nih.gov/geo/query/acc.cgi?acc=GSE218540).

### Gene set enrichment analysis (GSEA)

RNAseq data (*n* = 19–20) was first corrected using software packages developed in version 2.6.0 of Bioconductor and R version 2.10.1^34,35^ and adjusted for false discovery rate (FDR). The resulting data were then analyzed using the GSEA software developed by the Broad Institute as per their instructions^36^ using treatment group (NG, HG or HG + drugs) as a categorical label and the platforms Reactome, KEGG and Biocarta. Using a permutation test with 1,000 repetitions, the cutoff of significance level *p*-values was chosen as <0.05 for the significant pathways with any gene sets that had <10 genes and >500 genes being excluded to avoid inflated scores for small gene sets and inaccurate normalization for larger gene sets. From this analysis, genes were ranked based on their magnitude of the difference in expression between the phenotypic states. The key scores for this analysis were as follows: (1) Normalized Enrichment Score (NES): the degree to which a set of genes is overrepresented with the magnitude of the increment dependent on the correlation of the gene to the phenotype normalizing to the size of the gene set, (2) FDR corresponding to each NES to correct for gene set size and multiple hypotheses testing. An FDR ≤0.25 is recommended by the software developers to identify significantly associated gene sets.^37^ The gene sets with an FDR <0.25 were considered for the NG vs HG analysis and an FDR <0.3 for HG vs HG + drugs (as this was a smaller data set). As the final step, each pathway was classified based on its function as identified in Reactome and manually assigned to functional classes.

## Results

### A cell model of reversible β-cell dysfunction

**Figure 1.**
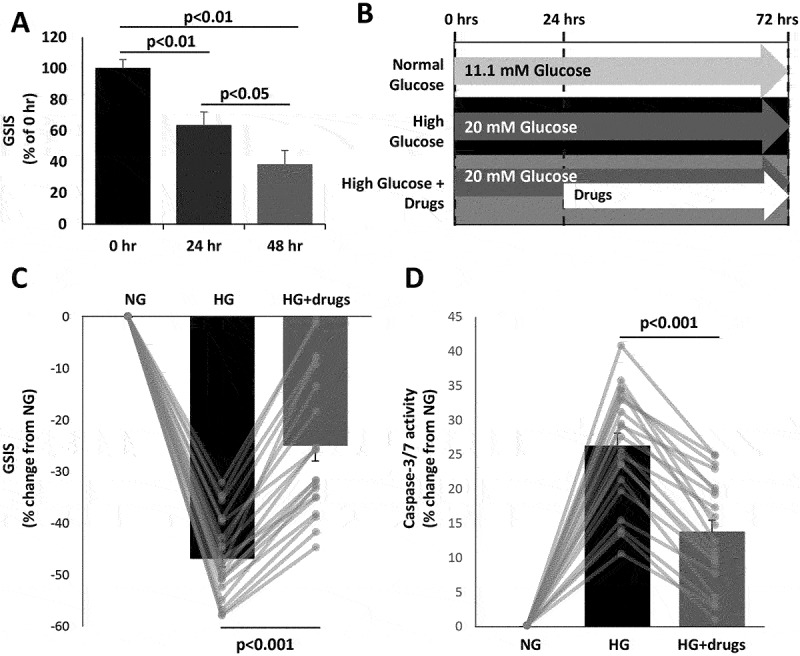
Induction and reversal of glucose induced β-cell dysfunction and reduced viability. (A) Glucose-stimulated insulin secretion (GSIS) in INS1E cells cultured in 20 mM glucose (high glucose) for 0, 24 or 48 h. *N* = 4 independent experiments. (B) Treatment protocol, INS1E cells cultured in 11.1 mM glucose for 72 h (normal glucose, NG), 20 mM glucose for 72 h (high glucose, HG) or 20 mM glucose for an initial 24 h and then 20 mM glucose plus drugs (250 μM metformin, 50 nM exendin-4 and 250 μM sodium salicylate) for 48 h (HG + drugs) with drugs refreshed after 24 h. (C) Change in glucose-stimulated insulin secretion (GSIS) and (D) change in apoptosis (caspase-3/7 activity) from NG, bars show average + SEM with individual experiments shown in gray lines. *N* = 20 independent experiments.

The ideal point at which to intervene with β-cell protective treatments would be when an individual is first diagnosed with T2D (or pre-diabetes) before they lose too much β-cell functional mass that the treatment would not be effective. Therefore, we aimed to use a cell model where early dysfunction has been induced, but some function still remains. INS-1E cells were cultured in HG (20 mM) to induce dysfunction. After 24 and 48 h in HG, GSIS was reduced by 38 ± 3.7% and 61 ± 9.6%, respectively (Figure 1a). The decline in function at 24 h mimics early dysfunction, while the progressive decline over the following 24 h mimics what would happen without treatment. Therefore, HG treatment for 24 h was selected as the optimal initial treatment prior to reversal treatment due to its ability to induce dysfunction yet not cause such overt damage that drug treatments would not be able to reverse the dysfunction.

Several drugs were screened for their ability to reverse dysfunction. The final cocktail and the concentrations used (50 nM exendin-4 and 250 μM NaS) were selected so that each drug had an intermediate effect on cell function and/or apoptosis and the effect of one drug would not be predominant (250 μM metformin). This was to ensure that the resultant pathway results would not simply represent the effects of one drug but be more representative of the functional state of the cells. Metformin, a common treatment for T2D acting on the liver to reduce glucose production, has also been shown to have a direct effect on β-cells and islets to increase viability and function.^38,39^ Exendin-4 is a GLP-1 receptor agonist that can directly increase insulin secretion and proliferation^40^ while NaS is an anti-inflammatory agent that has also been shown to have direct, positive effects on islets.^41^ Thus, while this combination of drugs is not routinely used in patients, each of the drugs acts via a different mechanism to protect β-cells. Therefore, by using the combination of these drugs the resultant pathway findings would be more likely to be representative of the overall function of the cells rather than showing the effect of a single drug.

The final cell model is represented in Figure 1b. After exposure of INS-1E cells HG for 72 h, cell function was reduced by 47 ± 1.7% and apoptosis was increased by 26 ± 1.9% compared to NG (Figure 1c and d, respectively). Treatment with 250 μM metformin, 50 nM exendin-4 and 250 μM NaS during the final 48 h of the HG treatment (HG + drugs) reversed the impairment in cell function by 49 ± 5.5% and apoptosis by 51 ± 4.5% compared to HG alone (Figure 1c and d, respectively). These results show that we successfully created a cell-based model where we induced and reversed β-cell dysfunction/reduced viability.

### Pathways altered with HG and reversed with drugs

**Figure 2.**
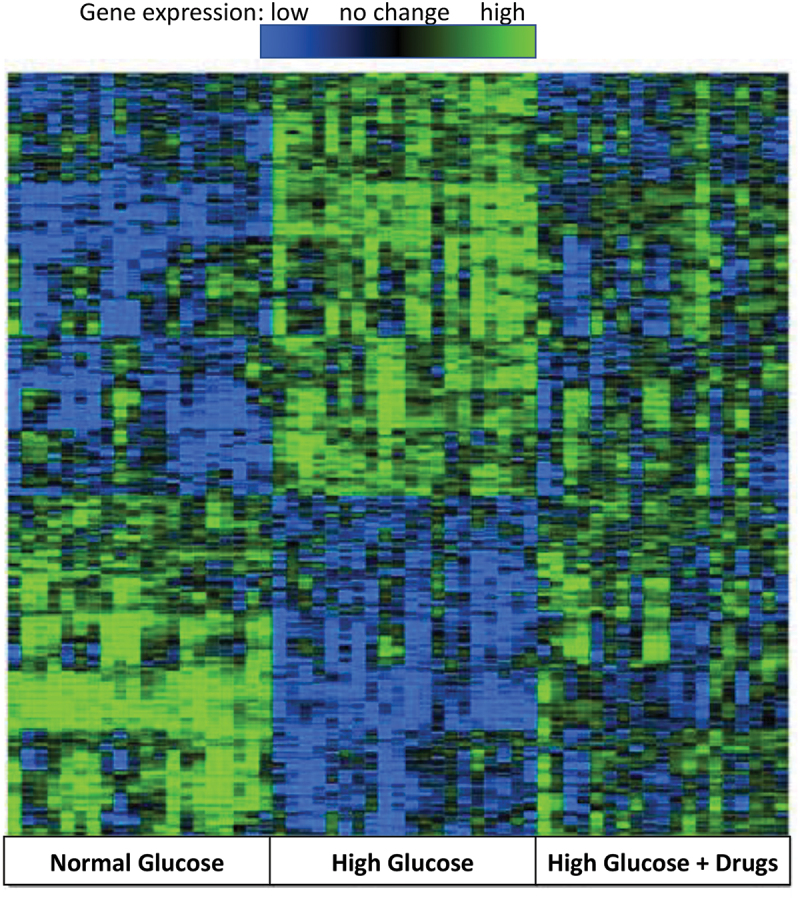
Overall gene expression changes in normal glucose, high glucose and high glucose + drugs INS-1E cells. Highest expression (green), lowest expression (blue) through not changed (black). Genes showing an interquartile range <0.25 are not shown. *N* = 20 independent experiments.

There were clear differences in overall gene expression in cells treated with HG compared to those treated with NG (Figure 2). When cells were treated with HG + drugs for the final 48 h to restore cell function, the overall gene expression changes were partially restored toward the levels in NG-treated cells (Figure 2).

**Figure 3.**
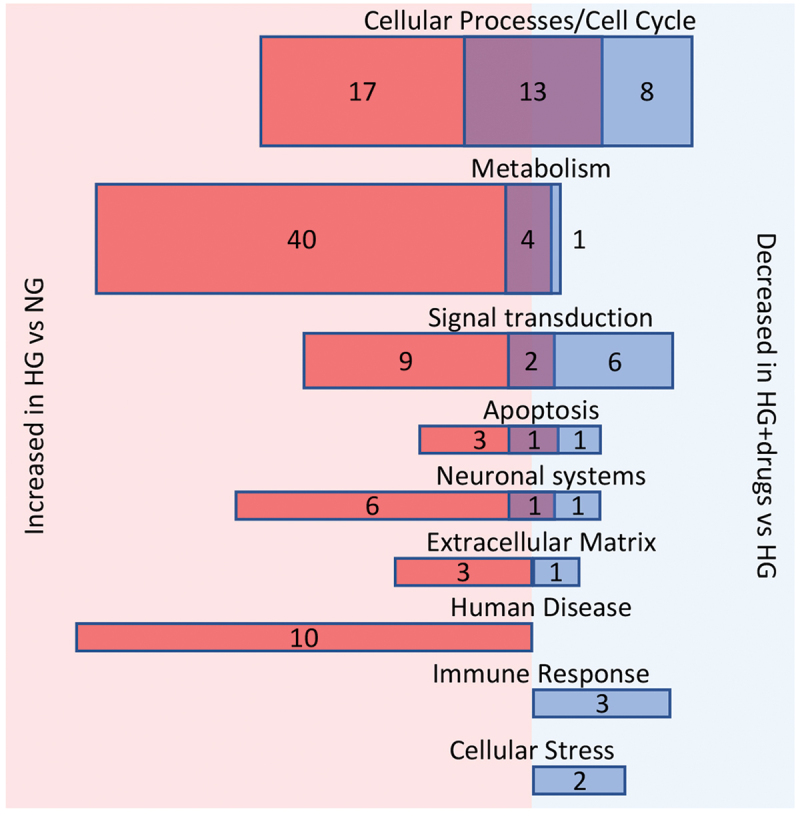
Pathways enriched in HG vs NG and/or HG + drugs vs HG. Number of significantly enriched pathways from gene set enrichment analysis (GSEA) in each functional pathway classification. Red, pathways increase in high glucose (HG) vs normal glucose (NG); Blue, pathways decreased in HG + drugs vs HG; Purple overlap, pathways both increased with HG (vs NG) and then decreased with drugs (vs HG alone). Size of the bars is representative of the number of pathways.

GSEA was then performed on all genes to allocate them into gene sets based on their known functions. No pathways were significantly reduced with HG treatment compared to NG, but 109 pathways were significantly increased (Supplemental Table 1). To get a better understanding of these gene set pathways, they were then mapped into functional classes. These 109 pathways could be mapped into seven functional classes: ‘cellular processes/cell cycle,’ ‘metabolism,’ ‘signal transduction,’ ‘apoptosis,’ ‘neuronal systems,’ ‘human diseases’ and ‘extracellular matrix’ (Figure 3). The majority of these pathways have been previously identified as playing a role in diabetes which validates this cell model and analysis for its relevance to human disease.

With drug treatment, no pathways were significantly increased while 44 pathways were significantly reduced with the successful drug cocktail treatment (HG + drugs compared to HG, Supplemental Table 2). These pathways were then mapped into functional classes revealing significant changes in genes involved in the control of several aspects of β-cell function such as, ‘cellular processes/cell cycle,’ *‘*metabolism,’ *‘*signal transduction,’ *‘*apoptosis,’ *‘*neuronal systems,’ *‘*extracellular matrix,*‘*immune responses’ and ‘cellular stress’ (Figure 3).

Of the 109 pathways enriched with HG and the 44 pathways downregulated by drug treatment, 21 pathways were common (Figure 3). There was an additional 88 pathways upregulated with HG but unchanged with drug treatment and 23 pathways unchanged with HG but downregulated with drug treatment (Figure 3).

#### Cellular processes/cell cycle pathways

**Figure 4.**
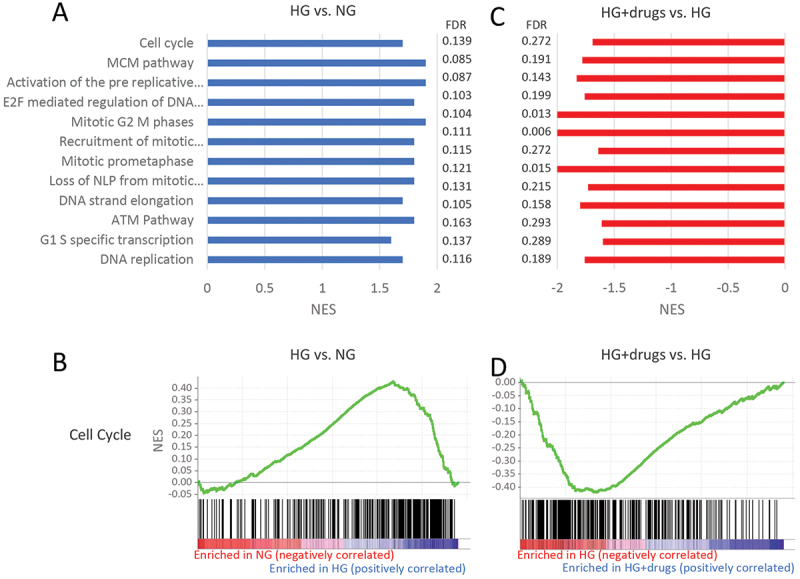
Pathways enriched in HG vs NG and HG + drugs vs HG. NG in the functional classification of *cellular processes/Cell cycle*. (A) Plots indicating pathway enrichment and (B) GSEA plots of representative gene sets. The NES indicates the degree to which the genes in a gene set were overrepresented at the extremes (top or bottom) of a ranked gene list. NES: Normalized Enrichment Score; FDR: false discovery rate; GSEA: gene set enrichment analysis; HG: high glucose.

Of the pathways identified, those involved in cellular processes were one of the largest components. 30 pathways were upregulated with HG and 21 downregulated with drugs (Supplemental Tables 1 and 2, respectively). Of these, 13 pathways were common. Figure 4a shows the NES of these pathways in HG vs low glucose (LG) and Figure 4c the NES in HG + drugs vs HG. The enrichment plots for the overall *Cell Cycle* pathway show that the vast majority of genes in this pathway were upregulated with HG vs LG (Figure 4b) and downregulated with drugs (Figure 4d).

#### Metabolism pathways

**Figure 5.**
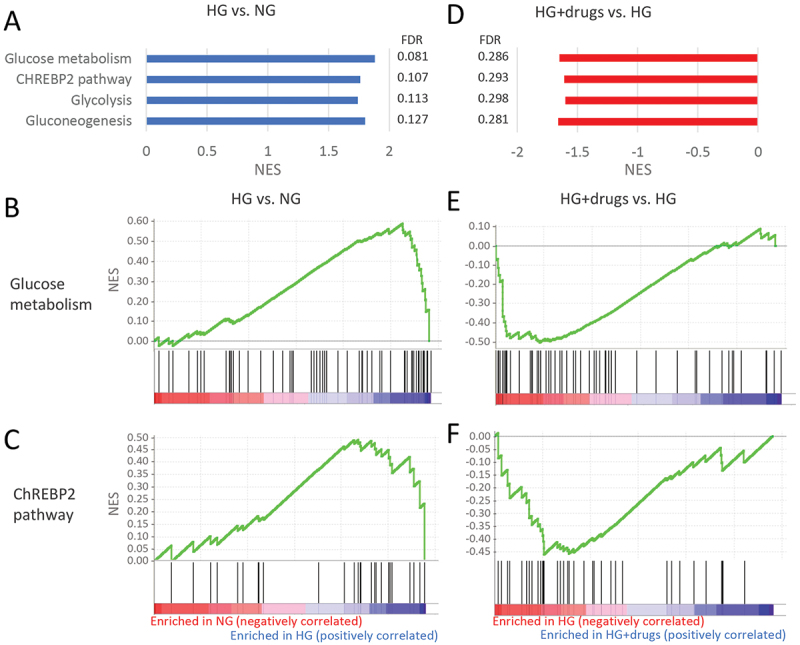
Pathways enriched in HG vs NG and HG + drugs vs HG in the functional classification of *metabolism*. (A) Plots indicating pathway enrichment and (B) GSEA plots of representative gene sets. The NES indicates the degree to which the genes in a gene set were overrepresented at the extremes (top or bottom) of a ranked gene list. NES: Normalized Enrichment Score; FDR: false discovery rate; GSEA: gene set enrichment analysis; HG: high glucose.

A total of 44 pathways involved in metabolism were upregulated with HG that were involved in a range of different processes (Supplemental Table 1). Treatment with drugs resulted in only five metabolism pathways being downregulated (Supplemental Table 2), four of which were reversing the effect of HG (Figure 5a and d). These pathways all focused on glucose/carbohydrate metabolism. The enrichment plots of the top two pathways, ‘Glucose Metabolism’ and ‘ChREBP2 pathway,*’* show that almost all genes were upregulated with HG (Figure 5b-c) and downregulated with drugs (Figure 5e-f).

#### Signal transduction pathways

**Figure 6.**
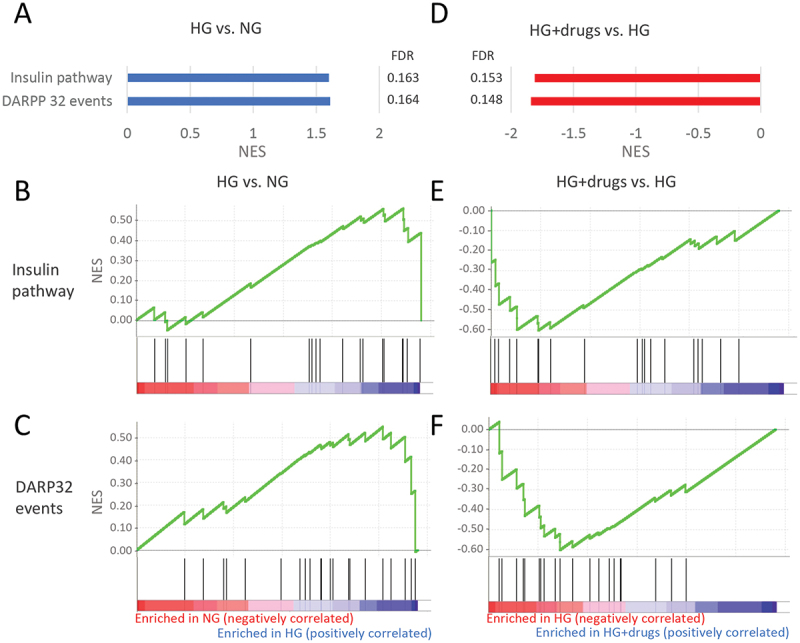
Pathways enriched in HG vs NG and HG + drugs vs HG. NG in the functional classification of *signal transduction*. (A) Plots indicating pathway enrichment and (B) GSEA plots of representative gene sets. The NES indicates the degree to which the genes in a gene set were overrepresented at the extremes (top or bottom) of a ranked gene list. NES: Normalized Enrichment Score; FDR: false discovery rate; GSEA: gene set enrichment analysis; HG: high glucose.

A total of 11 pathways involved in signal transduction were upregulated with HG (Supplemental Table 1). Eight pathways were downregulated with drugs (Supplemental Table 2) with two pathways in common, the ‘insulin pathway*’* and the *‘*DARPP 32 events pathway’ (Figure 6a and d). Again, almost all genes in these pathways were upregulated with HG (Figure 6b-c) and downregulated with drugs (Figure 6e-f).

#### Apoptosis

**Figure 7.**
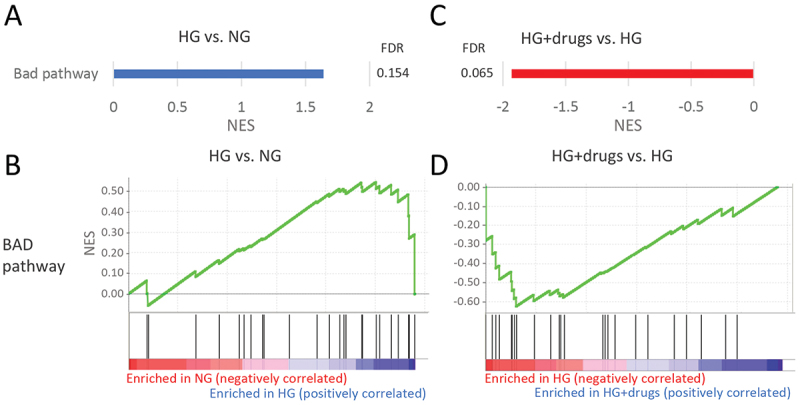
Pathways enriched in HG vs NG and HG + drugs vs HG. NG in the functional classification of *apoptosis*. (A) Plots indicating pathway enrichment and (B) GSEA plots of representative gene sets. The NES indicates the degree to which the genes in a gene set were overrepresented at the extremes (top or bottom) of a ranked gene list. NES: Normalized Enrichment Score; FDR: false discovery rate; GSEA: gene set enrichment analysis; HG: high glucose.

There were four pathways significantly enriched in HG in the functional classification of apoptosis (Supplemental Table 1). Two pathways were downregulated with HG + drugs (Supplemental Table 2) with one pathway (‘BAD*’*) in common (Figure 7a and c). Almost all genes in this pathway were upregulated with HG (Figure 7b) and downregulated with drugs (Figure 7d).

#### Neuronal systems

**Figure 8.**
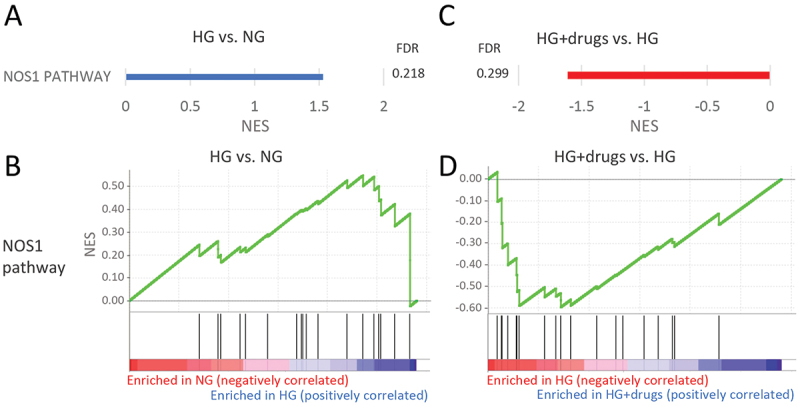
Pathways enriched in HG vs NG and HG + drugs vs HG. NG in the functional classification of *neuronal signaling*. (A) Plots indicating pathway enrichment and (B) GSEA plots of representative gene sets. The NES indicates the degree to which the genes in a gene set were overrepresented at the extremes (top or bottom) of a ranked gene list. NES: Normalized Enrichment Score; FDR: false discovery rate; GSEA: gene set enrichment analysis; HG: High Glucose.

There were seven pathways significantly enriched in HG in the functional classification of neuronal systems (Supplemental Table 1), two significantly downregulated with HG + drugs (Supplemental Table 2) with one in common (Figure 8a and c). Almost all genes in the *‘*NOS1 pathway*’* were upregulated with HG (Figure 8b) and downregulated with drugs (Figure 8d).

### Discussion

This study attempted to tackle the issue of finding appropriate drug targets for the protection of β-cells in a novel way. We suggest that obtaining a comprehensive view of transcriptional changes associated with β-cell dysfunction and restoration of function, we could identify those pathways that are druggable. Ultimately this information could contribute to drugs that improve β-cell function and survival. The analysis revealed several pathways associated with β-cell function which have important roles in various functions such as cellular processes, metabolism, signal transduction, apoptosis, neuronal systems and human diseases. Interestingly HG induced global upregulation of pathways associated with β-cell function but did not result in downregulation of pathways. Further, successful drug treatment only resulted in downregulation of pathways, some of which were reversing the effects of HG. Cellular processes and metabolism were the top functional class identified by HG vs NG analysis, whereas cellular processes and signal transduction were the top functional class regulated in the reversal group. Therefore, the reversal group showed a shift from the metabolic pathways toward signal transduction and other cellular functions.

Many of the gene sets identified by the HG vs NG GSEA analysis did not appear in the HG + drugs vs HG analysis. This is expected as the most important implication of this study was to select out the pathways that are simply regulated by HG from those that are more likely to play a role in the function and health of the β-cell. Thus, it is likely that only those pathways that are vitally relevant to the function and health of β-cell would have been reversed with the successful drug treatment. Moreover, this disparate transcriptional response could also imply that there may be some differences in the mechanisms that lead to the induction of β-cell dysfunction compared to the reversal of dysfunction, or in other words, the drug combination may have acted on the cells in a different way to improve cell function and viability. However, some of the pathways are also common in both analyses suggesting the drugs may had some direct effects to reverse pathways altered by HG.

### HG upregulation of cell cycle is reversed with drug treatment

*‘*Cellular Processes/Cell Cycle*’* was the most highly represented functional class among both analysis (HG vs NG and HG + drugs vs HG). The majority of these pathways are associated with cell division, proliferation and survival. Several of these pathways were upregulated in the HG phenotype which have previously known functions in β-cell proliferation and insulin secretion.^42–45^ The upregulation of these pathways suggests that β-cells are attempting to enter the cell cycle, possibly to expand mass to compensate for the demands of hyperglycemia. A similar effect is seen in response to an insulin resistant state such as obesity or pregnancy, where β-cell mass expands to adjust with the increased insulin demand partly due to β-cell proliferation.^46–48^ Multiple cell cycle pathways were restored with drugs suggesting that the increased demand may be partially restrained.

### HG upregulation of metabolism is partially reversed with drug treatment

The second major functional class identified by our study was *‘*Metabolism.*’* This is expected as β-cells are highly metabolically active and hyperglycemia has previously been shown to alter the expression of genes involved in multiple metabolic pathways from normal β-cell glucose metabolism.^26,49–52^ There were also several pathways upregulated by HG involved in gene expression (involving PGCA1 and RNA polymerase III) as well as overall RNA and protein metabolism. This is consistent with the large transcriptional changes that were observed in HG vs NG and also indicates an increase in protein production in response to these changes. This could also indicate increased protein synthesis for growth in response to HG.^53^ Importantly, there were increases in pathways involving glucose metabolism, as would be expected under HG conditions. Moreover, pathways associated with fatty acid and triglyceride biosynthesis and metabolism of lipids and lipoproteins, were also upregulated validating previous studies showing an adaptive response of chronic exposure of β-cells to HG by inducing glycolytic and lipogenic genes in conjunction with glycogen and triglyceride deposition, as well as increased anaplerosis and altered lipid partitioning.^54^

Surprisingly, compared to the number of metabolic pathways affected with, only a fraction of metabolic pathways were reversed with the drugs. This could suggest that the upregulation of several metabolic pathways in the HG vs LG group associated with metabolism could be mainly due to the orchestration of general effects of HG rather than its actual role in the pathology of the disease. Thus, drugs that target these pathways may not be greatly beneficial in treating β-cell dysfunction. However, the reversal of some metabolic pathways with HG + drugs treatment suggests that the drugs that can improve β-cell function could eventually restore the dysfunctionality in metabolism caused by HG. This is crucial information as to our knowledge no previous studies have demonstrated the effect of the reversal of dysfunction on these pathways. Thus, although metabolism has been demonstrated to be uniquely important in β-cell function,^26^ our data suggest that there may be other functional pathways which may play a greater role in improving β-cell function than the previously appreciated metabolic pathways. Moreover, Webb et al., in their study to identify genes involved in insulin secretion and synthesis by pancreatic β-cells, suggested that glucose metabolism may play a lesser role in improving β-cell function,^26^ which concurs with our current findings. Overall, while several studies have focused on identifying targets to modify changes in the metabolic pathways in diabetes,^55^ our study demonstrates that targeting metabolic pathways may not be an ideal way to improve β-cell function and viability.

### HG upregulation of signal transduction is partially reversed with drug treatment

Our study demonstrated that several ‘signal transduction pathways’ were also upregulated with HG treatment mainly involved in β-cell insulin secretion, cell differentiation, proliferation and survival. The upregulation of these pathways in our HG treated group suggests a possible attempt of these cells to maintain appropriate β-cell mass, insulin production and survival in response to hyperglycemia. However, only ‘Insulin Pathway’ and ‘DARPP32 Events’ were reversed with our drug treatment. The reversal of these pathways with our drug treatment suggests that these pathways are druggable and can be possible targets to improve β-cell and viability. The insulin pathway is not just crucial for insulin exocytosis but is also a positive regulator of β-cell mass and is equally crucial for protection against β-cell apoptosis.^56^ Similarly, *‘*DARPP 32 events pathway is a key component of the insulin signaling pathway which is responsible for bridging the initial insulin-stimulated phosphorylation cascade with the ultimate dephosphorylation of insulin sensitive substrates.^57^ However, there is little information available for the DARPP32 pathway in β-cells. DARPP32 (dopamine- and cyclic-AMP-regulated phosphoprotein) is a neuronal phosphoprotein, which is an inhibitor of protein phosphatase 1 (PP1). The presence of DARPP32 in β-cell has been previously studied by Lilja et al., where they indicated its potential role in the regulation of PP1 activity in pancreatic β-cell stimulus-secretion coupling.^58^ However, it is still unclear how this plays a role in β-cell function. Hence our study proposes a likely role of DARPP32 events pathway in the restoration of β-cell function which warrants further investigation.

### HG upregulation of the pro-apoptotic BAD pathway is reversed with drug treatment

Our study also showed that cellular stress and apoptosis pathways were upregulated under the HG condition. These pathways have been heavily studied in β-cell dysfunction.^59–63^ In T2D, β-cell mass is progressively decreased due to an increase in apoptosis.^59,63–65^ Hence, identification of pathways involved in cellular stress and apoptosis and targeting them with drugs could improve cell viability and thereby delay the progression of T2D. This study identified one pathway which was reversed with drug treatment, ‘BAD,’ suggesting that this pathway could perhaps be a potential target for improving cell viability. BAD, a proapoptotic Blc2 protein, has been considered as a multifunctional protein which could modulate both cell survival and cell death pathways.^66^ In pancreatic β-cells, BAD has been shown to have physiological role in the regulation of insulin secretion and β-cell mass.^67^ However, under HG condition, *Bad* expression is significantly upregulated.^68^ Moreover, evidence suggests that BAD plays an important role in HG-induced β-cell death in cultured human islets.^69^ The reversal of the BAD pathway with our drug treatment possibly suggests the druggability of the pathway for improving β-cell health.

### HG upregulation of neuronal system, NOS1 pathway, is reversed with drug treatment

Lastly, our study showed the NOS1 pathway was significantly upregulated with HG and successfully reversed with the drug treatment. NOS1 (also known as nNOS) has previously shown to have important role in normal β-cell function.^70^ Inhibition of NOS1 has shown to increase ER stress and activate JNK, therefore activation of NOS could protect β-cells from stress and apoptosis induced by glucolipotoxicity.^65^ Hence, upregulation of *‘*NOS1 pathway’ with HG in our study could represent an adaptive response to glucotoxicity. This is supported by a previous study that demonstrated that NOS1 activation in glucolipotoxic condition is an adaptive response to protect β-cells from stress and apoptosis.^65^ Hence, modulation of the NOS1 pathway could possibly be beneficial for restoring β-cell viability/health.

## Conclusions

Our GSEA analysis between control groups (HG vs NG) identified many known pathways, which validated the applicability of our cell model to human disease and the directionality of our current study. This group is important in providing clues to the pathogenesis of human T2D and may be of predictive value in efforts aiming at conferring β-cell protection against apoptosis, impaired regenerative capacity, and functional suppression occurring in diabetes. However, the novelty of this study was the inclusion of the reversal group (HG + drugs) that could provide valuable information on the pathways which can actually be modified by and targeted with drugs. The most interesting findings of this study were the conflicting evidence to the previous studies that focus on the metabolic pathways to restore insulin secretion and β-cell function. Features we found to be associated with restoration of β-cell function do not directly link to the metabolic pathways, instead we believe the alteration in the metabolic pathways could be a separate effect due to the prolonged hyperglycemic condition.

Moreover, our study demonstrated the genome-wide integration of β-cell function at the level of transcript abundance and validates the efficacy of expression profiling in identifying gene sets/pathways involved in β-cell dysfunction and reversal of dysfunction. Overall, the described results demonstrate the widespread integration of important biological processes in β-cells and illustrates the complex dynamics of whole-cell adaptation to changes in glucose levels and the possibility of reversing this effect using drug treatments. Additionally, these findings demonstrating reversible pathways that are associated with resorted β-cell function may provide clues to the pathogenesis and possible drug targets for human T2D.

## Supplementary Material

Supplemental Material
