# Supplementary material for: Identification of reversible and druggable pathways to improve beta-cell function and survival in Type 2 diabetes
Source: Islets. 2023 Jan 29;15(1):2165368. doi: 10.1080/19382014.2023.2165368 (PMC9888462; doi:10.1080/19382014.2023.2165368)
Supplement: Supplemental Material [file KISL_A_2165368_SM0982.zip › supplementary/Supplemental_Tables.docx]

| **Supplemental Table 1. Pathways Upregulated with HG vs. LG by functional classification** | | | | |
| --- | --- | --- | --- | --- |
| Pathway | Functional classification | SIZE | HG vs LG | |
|  |  |  | NES | FDR |
| **Cell Cycle** | | | | |
| G2/M CHECKPOINTS | Cell cycle | 39 | 1.9 | 0.082 |
| MCM pathway | Cell cycle/mitotic DNA replication | 18 | 1.9 | 0.085 |
| Activation of the pre replicative complex | Cell cycle/mitotic DNA replication | 29 | 1.9 | 0.087 |
| Mitotic G2 M phases | Cell cycle/mitotic G2-G2/M phases | 63 | 1.9 | 0.104 |
| ATM PATHWAY | Cell cycle | 19 | 1.8 | 0.105 |
| G1 PATHWAY | Cell cycle | 26 | 1.8 | 0.098 |
| E2F mediated regulation of DNA replication | Cell cycle/DNA replication | 31 | 1.8 | 0.103 |
| Recruitment of mitotic centrosome proteins and complexes | Cell cycle | 49 | 1.8 | 0.111 |
| Mitotic prometaphase | Cell cycle/ mitotic | 71 | 1.8 | 0.115 |
| Loss of NLP from mitotic centrosomes | Cell cycle/mitotic | 42 | 1.8 | 0.121 |
| Cell cycle | Cell cycle | 320 | 1.7 | 0.139 |
| DNA strand elongation | Cell cycle/mitotic | 29 | 1.7 | 0.131 |
| DNA replication | DNA replication | 170 | 1.7 | 0.137 |
| GH pathway | Cellular metabolism, mitogenesis and differentiation | 26 | 1.7 | 0.116 |
| SBASIGIN INTERACTIONS | Cell interactions | 22 | 1.7 | 0.134 |
| ARAP PATHWAY | vesicular trafficking | 15 | 1.7 | 0.117 |
| MITOTIC M G1 PHASES | Cell cycle/mitotic | 151 | 1.6 | 0.169 |
| RACCYCD PATHWAY | Cell cycle | 24 | 1.6 | 0.175 |
| G1 PHASE | Cell cycle | 29 | 1.6 | 0.176 |
| APC C CDC20 MEDIATED DEGRADATION OF CYCLIN B | Cell cycle/mitotic | 18 | 1.6 | 0.19 |
| G1 S specific transcription | Cell cycle/mitotic | 16 | 1.6 | 0.163 |
| APC CDC20 MEDIATED DEGRADATION OF NEK2A | Cell cycle/mitotic | 19 | 1.5 | 0.2 |
| FOCAL ADHESION | cell proliferation, differentiation, survival gene expression | 167 | 1.5 | 0.207 |
| CREB PATHWAY | Cell proliferation | 26 | 1.5 | 0.211 |
| G2 PATHWAY | Cell cycle | 20 | 1.5 | 0.216 |
| PHOSPHORYLATION OF THE APC C | Cell cycle/mitotic | 16 | 1.5 | 0.229 |
| G0 AND EARLY G1 | Cell cycle | 19 | 1.5 | 0.231 |
| INHIBITION OF THE PROTEOLYTIC ACTIVITY OF APC C | Cell cycle | 16 | 1.5 | 0.231 |
| KINESINS | Membrane transport | 20 | 1.5 | 0.249 |
| TRANSCRIPTIONAL REGULATION OF WHITE ADIPOCYTE DIFFERENTIATION | Adipogenesis/adipocyte differentiation | 60 | 1.48 | 0.231 |
| **Metabolism** | | | | |
| GLUCOSE TRANSPORT | Metabolism | 32 | 1.93 | 0.132 |
| PGC1A PATHWAY | Energy metabolism and homeostasis | 21 | 1.92 | 0.083 |
| Glucose metabolism | Metabolism of glucose | 58 | 1.88 | 0.081 |
| GLYCOLYSIS GLUCONEOGENESIS | Metabolism of glucose | 48 | 1.86 | 0.091 |
| TRIGLYCERIDE BIOSYNTHESIS | Metabolism of lipids | 37 | 1.83 | 0.102 |
| Gluconeogenesis | Metabolism of carbohydrate | 28 | 1.8 | 0.127 |
| FATTY ACYL COA BIOSYNTHESIS | Metabolism of lipids | 18 | 1.79 | 0.13 |
| PROTEIN FOLDING | Metabolism of proteins | 45 | 1.79 | 0.132 |
| GLUTATHIONE METABOLISM | Metabolism/ Oxidative stress | 46 | 1.78 | 0.125 |
| SYNTHESIS OF PA | Metabolism of lipids | 24 | 1.77 | 0.105 |
| CHREBP2 pathway | Metabolism of carbohydrate | 38 | 1.76 | 0.107 |
| Glycolysis | Metabolism of carbohydrate | 25 | 1.74 | 0.113 |
| PENTOSE PHOSPHATE PATHWAY | Metabolism | 22 | 1.72 | 0.118 |
| FATTY ACID TRIACYLGLYCEROL AND KETONE BODY METABOLISM | Fatty acid, triacylglycerol, and ketone body metabolism | 151 | 1.72 | 0.114 |
| METABOLISM OF NON CODING RNA | Metabolism of non-coding RNA | 41 | 1.71 | 0.119 |
| TRANSPORT OF MATURE MRNA DERIVED FROM AN INTRONLESS TRANSCRIPT | Metabolism of RNA | 28 | 1.69 | 0.124 |
| LINOLEIC ACID METABOLISM | Metabolism of amino acids | 16 | 1.67 | 0.139 |
| PYRUVATE METABOLISM | Metabolism of carbohydrate | 34 | 1.66 | 0.141 |
| PROCESSING OF CAPPED INTRONLESS PRE MRNA | Metabolism of RNA | 19 | 1.65 | 0.152 |
| METABOLISM OF LIPIDS AND LIPOPROTEINS | Metabolism of lipids and lipoproteins | 417 | 1.64 | 0.156 |
| PHASE II CONJUGATION | Metabolism/biological oxidations | 56 | 1.62 | 0.168 |
| CITRATE CYCLE TCA CYCLE | Metabolism of carbohydrates | 29 | 1.62 | 0.168 |
| METABOLISM OF RNA | Metabolism of RNA | 233 | 1.61 | 0.162 |
| GLYCEROPHOSPHOLIPID BIOSYNTHESIS | Metabolism of lipids | 72 | 1.61 | 0.163 |
| PREFOLDIN MEDIATED TRANSFER OF SUBSTRATE TO CCT TRIC | Metabolism of proteins | 22 | 1.61 | 0.165 |
| POST CHAPERONIN TUBULIN FOLDING PATHWAY | Metabolism of proteins | 15 | 1.6 | 0.166 |
| RNA POL III TRANSCRIPTION INITIATION FROM TYPE 2 PROMOTER | Gene expression | 20 | 1.56 | 0.19 |
| REGULATION OF GLUCOKINASE BY GLUCOKINASE REGULATORY PROTEIN | Metabolism of carbohydrate | 23 | 1.56 | 0.19 |
| FORMATION OF TUBULIN FOLDING INTERMEDIATES BY CCT TRIC | Metabolism of proteins (Chaperone mediated protein folding) | 18 | 1.56 | 0.19 |
| METABOLISM OF CARBOHYDRATES | Metabolism of carbohydrate | 202 | 1.55 | 0.199 |
| GLUTATHIONE CONJUGATION | Metabolism/biological oxidations | 22 | 1.55 | 0.192 |
| METABOLISM OF MRNA | Metabolism of mRNA | 194 | 1.53 | 0.212 |
| PPARA ACTIVATES GENE EXPRESSION | Metabolism of lipids | 89 | 1.52 | 0.214 |
| CHOLESTEROL BIOSYNTHESIS | Metabolism of lipids | 21 | 1.52 | 0.218 |
| TRANSLATION | Metabolism of proteins | 140 | 1.52 | 0.213 |
| ALPHA LINOLENIC ACID METABOLISM | Metabolism of amino acids | 15 | 1.52 | 0.211 |
| GLYCEROPHOSPHOLIPID METABOLISM | Metabolism of lipids | 66 | 1.51 | 0.21 |
| ACTIVATION OF THE MRNA UPON BINDING OF THE CAP BINDING COMPLEX AND EIFS AND SUBSEQUENT BINDING TO 43S | Metabolism of proteins | 53 | 1.51 | 0.209 |
| NONSENSE MEDIATED DECAY ENHANCED BY THE EXON JUNCTION COMPLEX | Metabolism of RNA | 98 | 1.5 | 0.22 |
| PYRUVATE METABOLISM AND CITRIC ACID TCA CYCLE | Energy metabolism and homeostasis | 39 | 1.5 | 0.223 |
| SYNTHESIS AND INTERCONVERSION OF NUCLEOTIDE DI AND TRIPHOSPHATES | Metabolism of nucleotides | 16 | 1.49 | 0.23 |
| 3 UTR MEDIATED TRANSLATIONAL REGULATION | Metabolism of proteins | 100 | 1.49 | 0.228 |
| TCA CYCLE AND RESPIRATORY ELECTRON TRANSPORT | Metabolism | 108 | 1.48 | 0.234 |
| FORMATION OF THE TERNARY COMPLEX AND SUBSEQUENTLY THE 43S COMPLEX | Metabolism of proteins | 46 | 1.47 | 0.239 |
| **SIGNAL TRANSDUCTION** | | | | |
| INSULIN SIGNALLING PATHWAY | Insulin signalling | 122 | 1.77 | 0.109 |
| WNT PATHWAY | WNT signalling | 25 | 1.62 | 0.171 |
| REGULATION OF KIT SIGNALLING | SCF-KIT signalling | 15 | 1.62 | 0.171 |
| DARPP 32 events | GPCR signalling | 21 | 1.61 | 0.164 |
| Insulin pathway | Insulin signalling | 20 | 1.6 | 0.163 |
| EFFECTS OF PIP2 HYDROLYSIS | GPCR signalling | 18 | 1.56 | 0.189 |
| SHC1 EVENTS IN ERBB4 SIGNALLING | ERBB4 signalling | 19 | 1.52 | 0.212 |
| SIGNALLING BY SCF KIT | SCF-KIT signalling | 71 | 1.5 | 0.227 |
| SIGNALLING BY RHO GTPASES | RAS signalling | 90 | 1.49 | 0.228 |
| ERK MAPK TARGETS | NGF signalling | 19 | 1.48 | 0.233 |
| ALK Pathway | TGFB signalling | 32 | 1.46 | 0.248 |
| **APOPTOSIS** | | | | |
| ACTIVATION OF ATR IN RESPONSE TO REPLICATION STRESS | Stress response | 33 | 1.95 | 0.21 |
| Bad pathway | Apoptosis | 24 | 1.64 | 0.154 |
| INTRINSIC PATHWAY FOR APOPTOSIS | Apoptosis | 26 | 1.61 | 0.166 |
| P53 PATHWAY | Apoptosis | 16 | 1.47 | 0.238 |
| **NEURONAL SYSTEMS** | | | | |
| ACTIVATION OF NMDA RECEPTOR UPON GLUTAMATE BINDING AND POSTSYNAPTIC EVENTS | Neuronal systems | 35 | 1.48 | 0.233 |
| CREB PHOSPHORYLATION THROUGH THE ACTIVATION OF CAMKII | Neuronal systems | 15 | 1.52 | 0.216 |
| CREB PHOSPHORYLATION THROUGH THE ACTIVATION OF RAS | Neuronal systems | 26 | 1.7 | 0.118 |
| POST NMDA RECEPTOR ACTIVATION EVENTS | Neuronal systems | 32 | 1.47 | 0.242 |
| RAS ACTIVATION UOPN Ca2+ INFUX THROUGH NMDA RECEPTOR | Neuronal systems | 17 | 1.74 | 0.115 |
| SIGNALLING BY ROBO RECEPTOR | Axon guidance | 28 | 1.46 | 0.246 |
| NOS1 PATHWAY | Neuronal systems/inflammation | 20 | 1.53 | 0.218 |
| **EXTRACELLULAR MATRIX** | | | | |
| DEGRADATION OF THE EXTRACELLULAR MATRIX | Extracellular matrix organisation | 20 | 1.74 | 0.119 |
| ECM RECEPTOR INTERACTION | Extracellular matrix | 61 | 1.61 | 0.165 |
| INTEGRIN PATHWAY | Extracellular matrix/cell cycle | 36 | 1.58 | 0.176 |
| **HUMAN DISEASE** | | | | |
| INTERACTIONS OF VPR WITH HOST CELLULAR PROTEINS | Disease | 27 | 1.73 | 0.116 |
| NEP/NS2 INTERACTS WITH THE CELLULAR EXPORT MACHINERY | Disease | 23 | 1.72 | 0.118 |
| TRANSPORT OF RIBONUCLEOPROTEINS INTO THE HOST NUCLEUS | Disease | 23 | 1.68 | 0.134 |
| FACTORS INVOLVED IN MEGAKARYOCYTE DEVELOPMENT AND PLATELET PRODUCTION | Haemostasis | 85 | 1.64 | 0.154 |
| ALZHEIMERS DISEASE | Disease | 144 | 1.58 | 0.176 |
| PROXIMAL TUBULE BICARBONATE RECLAMATION | Haemostasis | 20 | 1.57 | 0.183 |
| INFLUENZA LIFE CYCLE | Disease | 125 | 1.56 | 0.187 |
| PATHOGENIC ESCHERICHIA COLI INFECTION | Disease | 48 | 1.52 | 0.214 |
| AUTOIMMUNE THYROID DISEASE | Disease | 16 | 1.47 | 0.241 |
| INFLUENZA VIRAL RNA TRANSCRIPTION AND REPLICATION | Disease | 95 | 1.46 | 0.248 |
| The columns represent; pathway name, functional class of the pathway, number of genes in the gene set after filtering out those genes not in the expression dataset, normalised enrichment score (NES) for the gene set and false discovery rate (FDR, <0.25 was considered significant). | | | | |

| **Supplemental Table 2. Pathways Downregulated with HG+drugs vs. HG by functional classification** | | | | |
| --- | --- | --- | --- | --- |
| Pathway | Functional classification | SIZE | HG+drugs vs HG | |
|  |  |  | NES | FDR |
| **Cell Cycle** | | | | |
| Recruitment of mitotic centrosome proteins and complexes | Cell cycle | 49 | -2.16 | 0.006 |
| Loss of NLP from mitotic centrosomes | Cell cycle/mitotic | 42 | -2.07 | 0.015 |
| Mitotic G2 M phases | Cell cycle/mitotic G2-G2/M phases | 63 | -2.06 | 0.013 |
| ASSOCIATION OF LICENSING FACTORS WITH THE PRE REPLICATIVE COMPLEX | Cell cycle/DNA replication | 12 | -1.97 | 0.044 |
| CHROMOSOME MAINTENANCE | Cell cycle | 74 | -1.83 | 0.14 |
| Activation of the pre replicative complex | Cell cycle/mitotic DNA replication | 29 | -1.83 | 0.143 |
| ATM PATHWAY | Cell cycle | 19 | -1.8 | 0.158 |
| MCM pathway | Cell cycle/mitotic DNA replication | 18 | -1.78 | 0.191 |
| E2F mediated regulation of DNA replication | Cell cycle/DNA replication | 31 | -1.76 | 0.199 |
| GH pathway | Cellular metabolism, mitogenesis and differentiation | 26 | -1.76 | 0.189 |
| UNWINDING OF DNA | cell cycle | 11 | -1.74 | 0.214 |
| DNA strand elongation | Cell cycle/mitotic | 29 | -1.73 | 0.215 |
| IGF1R PATHWAY | Cell survival and proliferation | 22 | -1.73 | 0.207 |
| Cell cycle | Cell cycle | 320 | -1.69 | 0.272 |
| INHIBITION OF REPLICATION INITIATION OF DAMAGED DNA BY RB1 E2F1 | Cell cycle | 10 | -1.69 | 0.274 |
| NGF PATHWAY | cell survival, growth and differentiation/neuronal | 12 | -1.68 | 0.263 |
| Mitotic prometaphase | Cell cycle/ mitotic | 71 | -1.64 | 0.272 |
| EXTENSION OF TELOMERES | Cell cycle | 26 | -1.64 | 0.28 |
| G1 S specific transcription | Cell cycle/mitotic | 16 | -1.61 | 0.293 |
| DNA replication | DNA replication | 170 | -1.6 | 0.289 |
| CELL CYCLE MITOTIC | Cell cycle | 270 | -1.59 | 0.297 |
| **Metabolism** | | | | |
| DEADENYLATION OF MRNA | Metabolism of RNA | 16 | -1.67 | 0.269 |
| Gluconeogenesis | Metabolism of carbohydrate | 28 | -1.66 | 0.281 |
| Glucose metabolism | Metabolism of glucose | 58 | -1.65 | 0.286 |
| CHREBP2 pathway | Metabolism of carbohydrate | 38 | -1.61 | 0.293 |
| Glycolysis | Metabolism of carbohydrate | 25 | -1.6 | 0.298 |
| **SIGNAL TRANSDUCTION** | | | | |
| ENDOSOMAL SORTING COMPLEX REQUIRED FOR TRANSPORT (ESCRT) | Vesicle mediated transport/membrane trafficking | 22 | -1.86 | 0.126 |
| DARPP 32 events | GPCR signalling | 21 | -1.84 | 0.148 |
| Insulin pathway | Insulin signalling | 20 | -1.81 | 0.153 |
| ERKS ARE INACTIVATED | Signal transduction/immune system signalling | 11 | -1.73 | 0.223 |
| SOS MEDIATED SIGNALLING | signal transduction/insulin receptor signalling | 14 | -1.71 | 0.239 |
| EGFR DOWNREGULATION | Signal transduction/EGFR signalling | 23 | -1.68 | 0.27 |
| NUCLEAR EVENTS KINASE AND TRANSCRIPTION FACTOR ACTIVATION | Signal transduction/Kinase cascade | 22 | -1.66 | 0.281 |
| CREB PATHWAY | Signal transduction | 26 | -1.59 | 0.294 |
| **APOPTOSIS** | | | | |
| DREAM PATHWAY | Apoptosis | 11 | -1.93 | 0.058 |
| Bad pathway | Apoptosis | 24 | -1.93 | 0.065 |
| **NEURONAL SYSTEMS** | | | | |
| NOS1 PATHWAY | Neuronal systems | 20 | -1.61 | 0.299 |
| CDK5 PATHWAY | Neuronal systems | 10 | -1.6 | 0.293 |
| **EXTRACELLULAR MATRIX** | | | | |
| GPCR PATHWAY | Extra cellular signals/cell signalling cascade | 32 | -1.64 | 0.287 |
| **IMMUNE** | | | | |
| 41BB PATHWAY | immune response | 12 | -1.68 | 0.263 |
| MAPK TARGETS NUCLEAR EVENTS MEDIATED BY MAP KINASES | Immune systems | 28 | -1.62 | 0.295 |
| GATA3 PATHWAY | immune systems | 12 | -1.62 | 0.299 |
| **CELLULAR STRESS** | | | | |
| NUCLEOTIDE EXCISION REPAIR | Cellular stress/ Genotoxic stress | 40 | -1.77 | 0.189 |
| CFTR PATHWAY | Cellular stress/oxidative stress | 11 | -1.65 | 0.287 |
| The columns represent; pathway name, functional class of the pathway, number of genes in the gene set after filtering out those genes not in the expression dataset, normalised enrichment score (NES) for the gene set and false discovery rate (FDR <0.30 was considered significant). | | | | |
